# Quantum Hydrodynamic Theory for Sub-Nanometer Gaps: Atomic Protrusions Govern Near-Field Enhancement and Tunneling Signatures

**DOI:** 10.3390/ma19050856

**Published:** 2026-02-25

**Authors:** Qihong Hu, Yiran Wang, Xiaoyu Yang, Dong Xiang

**Affiliations:** Institute of Modern Optics and Center of Single Molecule Sciences, Tianjin Key Laboratory of Micro-Scale Optical Information Science and Technology, Nankai University, Tianjin 300350, China2120250463@mail.nankai.edu.cn (X.Y.)

**Keywords:** sub-nanometer nanogap, atomistic protrusion, quantum hydrodynamic theory, surface plasmons, quantum effects

## Abstract

**Highlights:**

**What are the main findings?**

- Atomistic protrusions barely shift far-field resonances but strongly reshape hotspot nanofocusing.
- Near-field enhancement is set by the protrusion aspect ratio competing with the nonclassical charge response.
- QHT predicts a red-to-blue crossover with suppressed enhancement in the tunneling-relevant regime.
- Protrusion geometry tunes the onset and strength of the crossover and near-field suppression.

**What are the implications of the main findings?**

- Far-field spectra can be misleading proxies for nanoscale field confinement below 1 nm gaps.
- Atomic-scale morphology becomes a practical design knob for quantum plasmonic field control.
- It provides QHT-based rules to engineer stable, extreme hotspots in sub-nanometer nanogaps.

**Abstract:**

As nanofabrication advances toward atom-by-atom control of surface morphology, plasmonic electrodes and nanogap devices are being pushed into a regime where atomic-scale protrusions and sub-nanometer separations become accessible. In this extreme limit, classical electrodynamics becomes unreliable because it cannot capture quantum effects. To this end, we compute the optical response of metallic sub-nanometer nanogaps containing atomic-scale protrusions by employing quantum hydrodynamic theory (QHT), and benchmark the predictions against the classical local-response approximation (LRA). We revealed that atomic-scale variations in protrusion can leave the far-field scattering spectrum nearly unchanged while profoundly reshaping tnear-field nanofocusing. Upon a continuous decrease in the nanogap, QHT successfully predicts non-monotonic spectral evolution with a redshift-to-blueshift deflection point accompanied via a suppression of field enhancement, whereas LRA yields a continuous redshift and a monotonic increase in field enhancement. We further demonstrated that such an inflection point is tunable, as determined by the atomic morphology of the electrodes, which provide a theoretical foundation for the experimental observation of varied inflection points. These results provide a practical route to optically diagnose and engineer tunneling-enabled charge exchange and quantum-regulated nanofocusing in extreme plasmonic nanogaps, and offer design guidance for molecular-scale optoelectronic and nanophotonic devices.

## 1. Introduction

Nanoscale optoelectronics and nanophotonics have long been driven by the goal of engineering optical functionality at the ultimate limit, where individual atoms or molecules serve as active device elements [1]. Surface plasmons offer a powerful route toward this goal by compressing electromagnetic energy far below the diffraction limit, enabling extreme nanofocusing and strongly enhanced light–matter interactions in nanogap resonators, molecular junctions, and scanning-probe geometries [2,3,4,5,6,7,8,9,10,11,12,13,14,15,16,17,18,19,20,21,22,23]. Crucially, advances in nanofabrication techniques, specifically Mechanically Controllable Break Junctions (MCBJs) and Scanning Tunneling Microscope Break Junctions (STM-BJs), have made it possible to manipulate electrode separations with sub-Ångström precision. These techniques routinely access the quantum tunneling regime in realistic devices [1,16,24], providing an experimental platform for verifying the predicted effects. As junction control approaches sub-nanometer separations, the relevant “device region” is no longer a smooth metal boundary but an atomically corrugated interface decorated by adatoms and atomic-scale protrusions. In this regime, understanding the optical response of nanogaps with atomic-scale asperities becomes essential, because these features can directly determine charge localization, field confinement, and energy dissipation [25,26,27].

A widely used starting point for modeling plasmonic nanogaps is classical local-response approximation (LRA), in which the metal is described by a frequency-dependent but spatially local permittivity [17,28,29,30,31,32]. LRA remains effective at nanometer-scale separations, yet it breaks down in the deep sub-nanometer regime, where quantum surface effects become unavoidable [33,34,35]. The most conspicuous failure appears as junctions enter the tunneling-relevant regime: experiments have reported that continued gap compression can drive a redshift-to-blueshift crossover of the dominant scattering resonances, accompanied by a suppression (rather than a divergence) of the gap enhancement [36]. Beyond spectral shifts, atomic protrusions introduce an additional—and equally critical—challenge. Even at a fixed nominal electrode separation, atomic-scale variations in protrusion geometry can profoundly reshape the near-field nanofocusing landscape, which LRA tends to overconfine because it omits spill-out, nonlocal response, and Landau damping [26,37,38]. Time-dependent density-functional theory (TD-DFT) provides a reference description of plasmonic excitations [39,40] but becomes prohibitively expensive for mesoscale tips and extended electrodes [41,42,43]. Quantum hydrodynamic theory (QHT) offers an effective middle ground: within a continuum framework, it incorporates electron spill-out, nonlocal response, and Landau damping through self-consistent coupling between Maxwell’s equations and conduction-electron dynamics, enabling systematic exploration of geometry-dependent trends at a fraction of the cost of TD-DFT [37,38,44,45,46,47,48,49]. This makes QHT particularly suitable for interrogating protrusion-controlled junction responses in the deep sub-nanometer regime.

Here we employ QHT to address these questions in a tip–substrate nanogap containing atomic-scale protrusions, and we benchmark all predictions against the classical LRA baseline. We show that atomic-scale variations can leave the far-field scattering lineshape almost unchanged while strongly reshaping near-field nanofocusing. Upon gap compression at a fixed protrusion, QHT predicts a non-monotonic spectral evolution with a tunneling signature—a redshift-to-blueshift crossover—together with a suppression of gap enhancement, whereas LRA yields a continuous redshift and a monotonic increase in the enhancement. Finally, by mapping representative junction geometries, we demonstrate that the crossover separation is strongly tunable via atomic-scale morphology, establishing protrusion design as a practical handle to engineer tunneling-enabled optical signatures and quantum-regulated nanofocusing in extreme plasmonic nanogaps.

## 2. Methods

**Figure 1:**
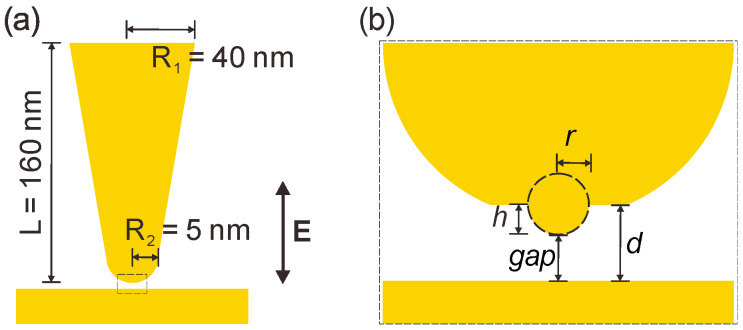
Geometry and parameter definitions for the tip–substrate nanogap. (**a**) Schematic of the tip–substrate geometry: a conical top electrode terminated by a spherical apex (apex radius 5 nm; cone base radius 40 nm; cone length 160 nm) facing a planar bottom electrode. (**b**) Enlarged view of the junction region highlighting an atomic-scale protrusion at the apex center. The protrusion is parameterized by its radius *r* and height *h*. The nominal electrode separation *d* is defined between the bottom of the spherical apex (excluding the protrusion) and the substrate surface, while the physical gap is defined as *gap* = *d* − *h* between the protrusion apex and the substrate surface.

All calculations are performed for a tip–substrate nanogap geometry (Figure 1). The top electrode is modeled as a conical tip terminated by a spherical apex (Figure 1a). The cone length is 160 nm with a base radius of 40 nm, and the apex radius is fixed at 5 nm throughout this work. The bottom electrode is modeled as a planar gold film treated as laterally infinite. A zoomed-in view of the junction region is shown in Figure 1b. An atomic-scale protrusion is introduced at the center of the spherical apex and is parameterized by (i) the protrusion radius *r* and (ii) the pulled-out height *h*. The nominal electrode separation *d* is defined as the distance from the bottom of the spherical apex (excluding the protrusion) to the planar substrate surface. The physical nanogap is then defined as *gap* = *d* − *h*, i.e., the distance from the protrusion apex to the substrate surface (Figure 1b). This parameterization allows us to (i) vary the protrusion geometry (*r*, *h*) at a fixed *d*, or (ii) scan *gap* while keeping the protrusion geometry fixed. Within the LRA, the optical response is obtained by solving the frequency-domain Maxwell equations using a local dielectric function for gold. We adopt a Lorentz–Drude description for Au [37],

$$\varepsilon_{\mathrm{Au}}\left(\omega \right)=\varepsilon_{\infty }-\frac{\omega_{p,1}^{2}}{\omega^{2}-\omega_{0,1}^{2}+i\omega \gamma_{1}}-\frac{\omega_{p,2}^{2}}{\omega^{2}-\omega_{0,2}^{2}+i\omega \gamma_{2}}.$$

The Drude term describes the free conduction electrons, where *ω*_p,1_ = 5.34 × 10^15^ rad/s, *ω*_0,1_ = 0 rad/s, and *γ*_1_ = 4.11 × 10^13^ rad/s are the plasma frequency, resonance frequency and damping rate, respectively. The Lorentz term accounts for the contribution of bound electrons, where *ω*_p,2_ = 4.85 × 10^15^ rad/s, *ω*_0,2_ = 4.30 × 10^15^ rad/s and *γ*_2_ = 7.48 × 10^14^ rad/s denote the resonance frequency, damping rate, and weighting factor of the *i*-th bound-electron resonance, respectively. The parameters were determined by fitting experimental data [50] in the wavelength range of 500–1200 nm to ensure accuracy in the optical regime of interest. The LRA calculations serve as the classical baseline and, by construction, do not capture quantum effects at sub-nanometer gaps.

To describe quantum effects relevant at molecular-junction length scales, we employ a linearized QHT in the frequency domain, which incorporates electron spill-out, nonlocal response, and Landau damping within a continuum framework. In this formulation, the electromagnetic field is solved self-consistently with a hydrodynamic description of the conduction-electron response. A convenient representation introduces an additional polarization field P describing the free-electron contribution, coupled to Maxwell’s equations through a linear constitutive relation in the frequency domain. In the QHT formulation, the electrodynamics can equivalently be expressed as [37,38,46,47,48,49]

$$\nabla \times \nabla \times E_{s}-\frac{\omega^{2}}{c^{2}}\varepsilon_{\mathrm{bg}}E_{s}-\omega^{2}\mu_{0}P-\frac{\omega^{2}}{c^{2}}\varepsilon^{*}\left(\omega \right)\left(E_{i}+E_{s}\right)=0,$$

$$\frac{en_{0}}{m_{e}}\nabla {\left(\frac{\delta G\left[n\right]}{\delta n}\right)}_{1}-\frac{e}{m_{e}}\nabla \cdot \left(\eta_{\sigma }\sigma \right)+\left(\omega^{2}+i\gamma_{1}\omega \right)P=-\varepsilon_{0}\omega_{p}^{2}\left(E_{s}+E_{i}\right).$$

Here **E_i_** (**E_s_**) and **P** are the incident (scattered) electric field and the polarization vector, respectively. c, ε_0_ and μ_0_ are the speed of light, the permittivity in a vacuum, and the permeability in a vacuum, respectively. m_e_ and e are the electron mass and charge. $\omega_{p}=\sqrt{e^{2}n_{0}/m_{e}\varepsilon_{0}}$ is the plasma frequency, with *n*_0_ being the ground-state electron density. The quantity (*δG*[*n*]/*δn*)_1_ can be obtained by adopting a first-order perturbation approach, where the perturbed density is taken as *n*(**r**) = *n*_0_(**r**) + *n*_1_(**r**), with *n*_1_ = ∇·**P**/e being a small (by assumption) first-order dynamic perturbation. Nonlocal damping or Landau damping is characterized by the viscoelastic tensor *σ*. The hydrodynamic closure relies on an internal-energy functional *G*[*n*] = *T*_TF_[*n*] + λ_vW_*T*_vW_[*n*] + *E*_xc_[*n*] for the electron gas, typically including Thomas–Fermi, von Weizsäcker, and exchange–correlation contributions, and introduces key model parameters such as the von Weizsäcker coefficient λ_vW_ and a nonlocal damping strength η_σ_. See Appendix A for the specific functional form. *ε*_bg_ = 1 is the permittivity of the background dielectric medium, and ε^*^ counts for the bound-electron response in the noble metal.

$$\varepsilon^{*}\left(\omega \right)=\varepsilon_{\infty }-\frac{\omega_{p,2}^{2}}{\omega^{2}-\omega_{0,2}^{2}+i\omega \gamma_{2}}-\varepsilon_{\mathrm{bg}}.$$

The ground-state electron density *n*_0_ is obtained self-consistently, which enables simulations for nanostructures of arbitrary geometry. The equation reads

$$\nabla \cdot \varepsilon \left(r\right)\nabla {\left(\frac{\delta G\left[n\right]}{\delta n}\right)}_{n=n_{0}}+e^{2}\left(n_{0}-n^{+}\right)=0,$$

with *n*^+^ = (4π*r*_s_^+^/3)^−1^ being the positive charge density for the uniform jellium background. In this work, we focus on gold with a Wigner–Seitz radius of *r*_s_^+^ = 3.12*a*_0_, where *a*_0_ is the Bohr radius. Unless otherwise stated, we follow the calibrated parameter strategy reported for optical-response calculations in ref. [46], where λ_vW_ and *η*_σ_ are fitted against DFT/TD-DFT benchmarks [46] (e.g., λ_vW_ = 0.70, *η*_σ_ = 7 for optical response, and λ_vW_ = 0.43, *η*_σ_ = 0 for the ground-state density profile). All of the above equations are implemented in COMSOL Multiphysics version 6.1 using weak form modules to solve for **E** and **P**.

To emulate an open domain, perfectly matched layers (PMLs) are applied at the outer boundaries. The substrate is embedded into the PML to approximate lateral infinity. The system is illuminated by a plane wave, with the electric-field polarization aligned along the tip axis (from the upper electrode to the lower electrode) to maximize the excitation of the gap mode. The mesh is locally refined in the nanogap region to resolve sub-nanometer geometric features and to ensure convergence of both near-field enhancement and scattering spectra. Concretely, we use a minimum element size of 0.03 nm in the vicinity of the protrusion apex and throughout the gap region (within a small refinement zone centered on the tip axis), while the remaining metal regions are meshed with a typical element size of 5 nm. The surrounding air domain is meshed more coarsely with a typical element size of 20 nm, and is smoothly transitioned to the fine gap mesh to avoid spurious numerical reflections. We extract the following observables: (i) the scattering spectrum, calculated from the outward Poynting flux through a fixed closed integration boundary; (ii) the maximum gap enhancement factor (EF) max(|**E**_s_|/|**E**_i_|), evaluated within a predefined sampling region adjacent to the protrusion apex; and (iii) near-field maps of |**E**_s_|/|**E**_i_| to visualize hotspot localization.

## 3. Results

### 3.1. Near–Far-Field Decoupling Induced by Atomistic Protrusions

**Figure 2:**
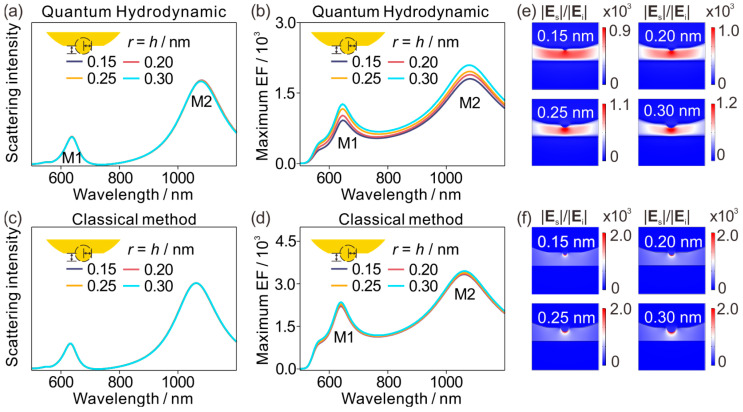
Far-field-robust but quantum-sensitive near-field enhancement for self-similar atomic protrusions. (**a**) QHT-computed scattering spectra for protrusions, the radius of identical to their height (0.15–0.30 nm) at a fixed nominal electrode separation, showing two nearly invariant resonances at 645 nm (M1) and 1080 nm (M2). (**b**) Corresponding maximum electric-field enhancement spectra inside the nanogap, exhibiting a monotonic increase as the protrusion scale increases. (**c**) LRA-computed scattering spectra for the same geometries, with resonances at 635 nm (M1) and 1060 nm (M2). (**d**) LRA maximum enhancement spectra, showing only weak sensitivity under self-similar scaling. (**e**) QHT enhancement maps for the four cases, revealing progressively tighter localization and stronger hotspot intensity at the protrusion apex as the protrusion is enlarged. (**f**) LRA enhancement maps for the same cases, remaining nearly unchanged with a hotspot pinned at the apex.

To isolate how atomic-scale protrusions reshape the optical response of metallic sub-nanometer nanogaps, we first consider a self-similar family of junctions in which the protrusion radius and height increase together, keeping a fixed aspect ratio (*r* = *h* = 0.15, 0.20, 0.25 and 0.35 nm). Figure 2a shows that the QHT scattering spectra are largely insensitive to self-similar changes in protrusion size: the M1 and M2 resonances remain nearly fixed and the lineshape is preserved. This insensitivity indicates that, within the explored atomic-scale range, the radiative response is dominated by global resonant charge oscillations that are weakly perturbed by the detailed morphology of a sub-nanometer asperity. The near field behaves very differently. Figure 2b plots the maximum field enhancement inside the gap region. Here QHT gives a clear trend: the enhancement grows as the self-similar protrusion becomes larger. Importantly, because the self-similar scan preserves the protrusion aspect ratio, this trend cannot be attributed to a changing “lightning rod” of the asperity. Instead, it reflects how QHT regulates charge accumulation and current flow at atomic length scales, where the induced response is redistributed within a thin interfacial region rather than being confined to a strictly local surface sheet. In this regime, nonclassical effects become decisive in setting the attainable hotspot intensity [38,47].

For comparison, the local classical baseline behaves as expected. Figure 2c shows that the LRA scattering spectra are likewise insensitive to the protrusion size. Figure 2d then shows that, under self-similar scaling, the LRA maximum enhancement changes only weakly—consistent with the fixed protrusion aspect ratio. The field maps sharpen the contrast. In Figure 2e (QHT), the hotspot increases in amplitude and becomes more spatially confined as the protrusion size increases, with the field progressively concentrating toward the apex. In Figure 2f (LRA), the hotspot remains pinned at the apex across all cases, with only minor variations. So, Figure 2 establishes a central message for metallic sub-nanometer nanogaps containing atomic-scale protrusions: atomic-scale asperities can leave the far-field scattering response nearly invariant, while the near-field enhancement becomes highly sensitive once quantum-corrected electrodynamics controls the interfacial response.

### 3.2. Aspect Ratio Control Versus Nonclassical Limitation of Nanofocusing

**Figure 3:**
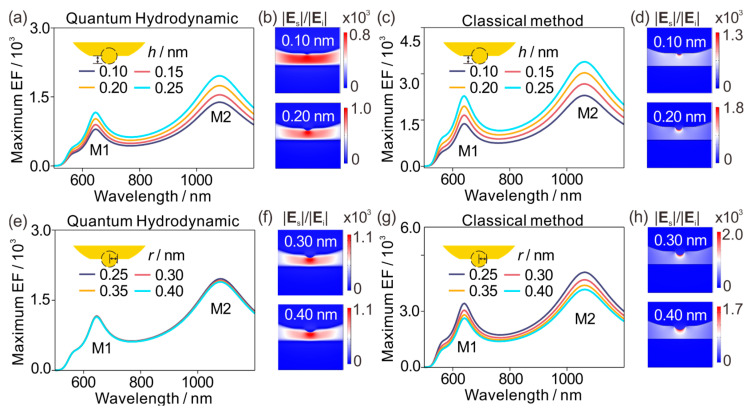
Aspect ratio modulation in metallic sub-nanometer nanogaps containing atomic-scale protrusions. (**a**) QHT maximum enhancement spectra for fixed *r* = 0.25 nm and varied *h* (0.10–0.25 nm). (**b**) Representative QHT enhancement maps for protrusion lengths of 0.10 nm and 0.20 nm, showing stronger confinement at the apex upon elongation. (**c**) LRA maximum enhancement spectra for the same height sweep. (**d**) Representative LRA enhancement maps for protrusion lengths of 0.10 nm and 0.20 nm. (**e**) QHT maximum enhancement spectra for fixed *h* = 0.25 nm and varied *r* (0.25–0.40 nm), showing an approximately radius-independent peak enhancement. (**f**) Representative QHT enhancement maps for protrusion radii of 0.30 nm and 0.40 nm. (**g**) LRA maximum enhancement spectra for the same radius sweep, decreasing monotonically as the apex is blunted. (**h**) Representative LRA enhancement maps for protrusion radii of 0.30 nm and 0.40 nm, showing reduced peak intensity at a larger radius.

We next decouple protrusion height and radius to explicitly tune the protrusion aspect ratio and thereby separate classical geometric focusing from quantum-limited nanofocusing. Figure 3 focuses on near-field observables only, since the scattering spectra are essentially unchanged in the corresponding parameter ranges (as already established above). We first hold the protrusion radius fixed at r = 0.25 nm and extend the protrusion height h (Figure 3a–d). This operation changes more than one thing at once: the effective confinement length scale along the gap direction shifts a bit, and, importantly, the protrusion becomes more elongated, so its aspect ratio increases. Figure 3a shows that QHT predicts a systematic rise in the maximum enhancement as the protrusion becomes more extended. Importantly, the enhancement increase is accompanied by a pronounced redistribution of the near-field, with stronger funneling and tighter localization toward the apex (Figure 3b). This evolution indicates that the enhancement increase is not only an amplitude effect but also a genuine strengthening of nanofocusing. The LRA benchmark is instructive here. Figure 3c shows that LRA also gives an increasing maximum enhancement with protrusion height. However, the spatial distribution remains essentially unchanged (Figure 3d). In Figure 3d, the hotspot pattern looks essentially the same for the two shown protrusion lengths: the enhancement is concentrated at the tip apex in both cases, with no obvious redistribution or tightening. In LRA, the protrusion primarily acts as a geometric concentrator, the effectiveness of which is governed by classical shape anisotropy (aspect ratio). In QHT, the same geometric tendency is present, but the achievable confinement and enhancement are concurrently regulated by quantum effects at the interface that redistribute induced current and electron density over a finite thickness (spill-out and nonlocal effect), thereby setting a nonclassical ceiling on “how sharply” the hotspot can be compressed. The self-similar control in Figure 2 provides a useful diagnostic reference. In that scan, radius and height are increased together, which also changes the physical gap in the same direction, yet the LRA enhancement barely responds. To rule out gap distance as the primary driver of these trends, we cross-compare junctions with identical physical gaps but distinct aspect ratios (Figure 2 and Figure 3). For a fixed height of *h* = 0.15 nm, increasing the radius from *r* = 0.15 nm to 0.25 nm causes the LRA enhancement to drop precipitously from 2215 to 1650. In striking contrast, the QHT enhancement remains virtually stable (910 to 900). A similar trend holds for *h* = 0.20 nm: blunting the tip reduces LRA enhancement by ~13% (2215 to 1650), whereas the QHT result remains perfectly invariant at 1000. That comparison strongly suggests that, in the present parameter window, the strong trends in Figure 3c,d are not dominated by gap reduction. Instead, they are dominated by the aspect-ratio of the protrusion: a more elongated feature is simply better at drawing induced charge toward the apex, and a local model can only express that classical geometric fact. QHT, on top of that, adds the second ingredient: nonclassical charge redistribution and the associated limitation on how sharply the hotspot can collapse.

We then fix the protrusion height h = 0.25 nm and increase the radius r, i.e., blunt the apex and reduce the aspect ratio from the other direction. Figure 3e shows the QHT result: the peak enhancement is nearly radius-independent over the explored range—an apparent saturation. Figure 3f supports the same reading in real space: the two representative maps look remarkably similar, both in confinement and intensity. In sharp contrast, the LRA results in Figure 3g,h display the expected classical monotonicity: the peak enhancement decreases as the protrusion becomes blunter (Figure 3g), while the hotspot remains pinned at the apex but steadily weakens in intensity (Figure 3h). This divergence between QHT and LRA is naturally explained by the two competing ingredients that control near-field enhancement in metallic sub-nanometer gaps: (1) classical geometric charge collection, governed primarily by the protrusion aspect ratio and curvature, which sets the tendency for field concentration; (2) quantum limitation of charge compression, arising from interfacial nonlocal response, electron spill-out, and Landau damping channels that delocalize induced current and electron density over a finite interfacial region, thereby suppressing unbounded geometric focusing. For QHT blunting, the apex weakens classical lightning-rod focusing, but it also relaxes the severity of quantum smoothing and high-gradient damping, which are strongest for extremely sharp, atomic-scale features. The net result is a weak dependence of the attainable hotspot intensity on curvature at fixed height, a behavior that is absent by construction in a purely local classical model.

Across Figure 2, the far-field scattering response remains nearly invariant under sub-nanometer variations in protrusion morphology, confirming that radiative resonances are controlled primarily by the global electrode geometry rather than by atomic-scale details. The near-field enhancement, by contrast, is governed by two dominant factors: First, there is a classical geometric lever; the protrusion aspect ratio controls how effectively induced charge is collected and funneled into the apex region. The LRA largely lives on this lever, and that is why it responds strongly when the aspect ratio is changed (Figure 3) but only weakly when self-similarity is preserved (Figure 2). Second, quantum effects impose a nonclassical constraint on hotspot compression. QHT captures interfacial charge and current redistribution through nonlocal response, spill-out and Landau damping, which become increasingly relevant as fields acquire large spatial gradients. These effects not only modify the peak enhancement but can also qualitatively change how the hotspot is spatially formed—most clearly evidenced by the QHT field-map evolution in Figure 3b and the curvature-induced saturation in Figure 3e,f, neither of which is reproduced by LRA.

### 3.3. Tunneling-Relevant Regime: Red-to-Blue Spectral Crossover and Field Suppression in QHT

**Figure 4:**
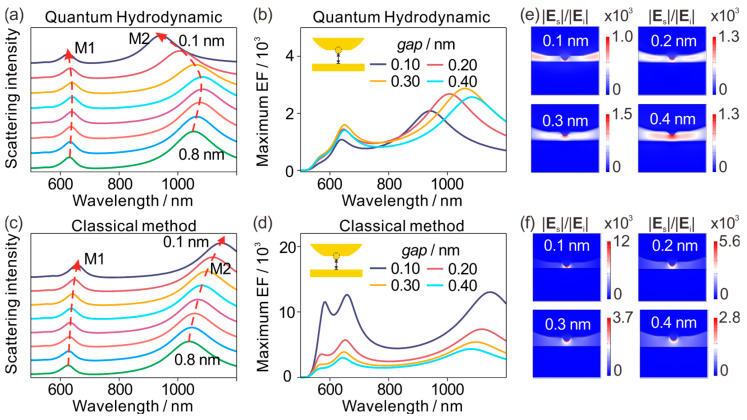
Gap-dependent red-to-blue crossover and non-monotonic near-field response at fixed protrusion geometry. The protrusion geometry is fixed (*r* = *h* = 0.25 nm). (**a**) QHT-computed scattering spectra for a gap scan from 0.8 nm to 0.1 nm, showing a non-monotonic evolution in which the resonances redshift upon initial compression (0.8–0.4 nm) and blueshift at smaller gaps (0.4–0.1 nm), producing a red-to-blue crossover with a turning point near *gap* = 0.4 nm. The dashed arrows indicate the change trends of the peak positions, and different colors of lines indicate the gap sizes varied from 0.8 nm to 0.1 nm. (**b**) QHT-predicted maximum EF inside the gap for selected narrow gaps (0.4, 0.3, 0.2, and 0.1 nm), increasing to about 0.3 nm and decreasing at 0.2 nm and below. (**c**) LRA scattering spectra for the same gap scan, exhibiting a continuous redshift with decreasing gap and no crossover. (**d**) LRA-predicted maximum EF inside the gap, increasing monotonically as the gap is reduced. (**e**) Representative QHT enhancement maps for selected gaps, showing suppression of gap-localized confinement at ultranarrow separations while a hotspot remains near the protrusion. (**f**) Representative LRA enhancement maps for the same gaps, with an apex-pinned hotspot that intensifies monotonically as the gap decreases.

In the preceding section, we showed that in metallic sub-nanometer nanogaps containing atomic-scale protrusions, near-field nanofocusing is highly susceptible to atomic-scale geometry, whereas the far-field scattering lineshape can remain nearly unchanged when the nominal electrode separation is held fixed. Here, we instead vary the gap by translating the electrodes. We first examine the gap dependence at a fixed protrusion geometry, mimicking a progressive compression cycle. In Figure 4, the protrusion radius and protrusion height are both fixed at 0.25 nm, while the gap (defined as the distance from the protrusion apex to the bottom electrode) is reduced from 0.8 nm to 0.1 nm. Figure 4a shows the QHT-computed scattering spectra for the gap scan from 0.8 nm down to 0.1 nm. Both resonances display a non-monotonic trajectory: from 0.8 nm to about 0.4 nm, the resonances redshift, whereas from about 0.4 nm down to 0.1 nm, they blueshift, producing a clear red-to-blue crossover with a turning point near a gap of roughly 0.4 nm. In this work, we use this red-to-blue crossover as the tunneling signature in scattering, i.e., an experimentally accessible spectral marker indicating that the junction has entered the tunneling-relevant, quantum-regulated coupling regime. Mechanistically, this spectral reversal originates from the spatial overlap of ground-state electron density tails (spill-out) across the junction. This non-classical electron distribution allows charge transfer to occur before the geometrical gap closes. This reversal is a characteristic signature of extreme confinement in which the junction response is no longer governed solely by a classical capacitive picture, but becomes regulated by quantum and nonlocal electron dynamics. The near-field response follows the same logic. Figure 4b summarizes the maximum field enhancement inside the gap for selected narrow gaps (0.4, 0.3, 0.2, and 0.1 nm). The enhancement increases as the gap is reduced from 0.4 nm to about 0.3 nm, but then decreases as the junction is compressed further to 0.2 nm and below. QHT therefore predicts that nanofocusing does not increase without bound: once the separation enters the deep sub-nanometer regime, the gap-localized hotspot is progressively suppressed.

The classical baseline is qualitatively different. Figure 4c shows the LRA scattering spectra under the same gap scan: both resonances redshift continuously as the gap is reduced, with no crossover. Figure 4d shows that the LRA maximum enhancement increases monotonically as the gap shrinks, as expected for an increasingly strong local capacitive confinement. For gaps below 0.4 nm, the LRA near-field spectra (Figure 4d) exhibit distinct splitting at ~580 nm for gaps below 0.4 nm, a feature not observed in far-field scattering (Figure 4c). This peak corresponds to a higher-order multipolar resonance that is highly localized at the protrusion apex. In the classical limit, the lack of a physical mechanism to limit field concentration leads to the abnormal amplification of such modes. However, in the QHT model, this splitting is entirely suppressed due to the nonlocal screening and electron spill-out (Figure 4b). This comparison rules out the LRA as a viable tool for deep sub-nanometer junctions. At these scales, LRA is not merely quantitatively inaccurate; it is missing the essential physics that controls both the spectral reversal and the near-field suppression, because it omits quantum effects that become decisive at sub-nanometer distances. The field maps visualize the same divergence. Figure 4e (QHT) shows that although a hotspot remains associated with the protrusion, the gap-localized confinement does not keep tightening indefinitely as the separation approaches 0.1–0.2 nm; instead, the gap-region enhancement weakens, consistent with the reduction in the peak enhancement in Figure 4b. Figure 4f (LRA), by contrast, shows an apex-pinned hotspot that intensifies monotonically with decreasing gap, mirroring the monotonic trend in Figure 4d. Taken together, Figure 4 establishes two model-dependent signatures: QHT predicts a robust red-to-blue crossover and a non-monotonic near-field response with suppression at ultranarrow gaps, whereas LRA yields a purely monotonic redshift and an unbounded enhancement increase upon compression. In the present deep sub-nanometer regime—and in light of the earlier protrusion results—LRA should therefore be regarded as qualitatively unreliable for both scattering spectra and near-field enhancement. We therefore use QHT alone in the following geometry-mapping study.

### 3.4. Geometry-Tunable Onset of the Tunneling-Relevant Crossover via Atomistic Protrusion Size

**Figure 5:**
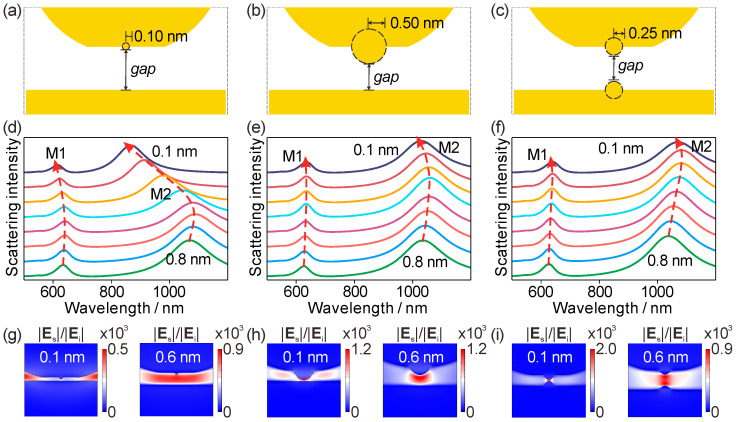
Geometry-tunable turning-point gap in QHT. (**a**–**c**) Three representative junction geometries: Structure I, single protrusion with *r* = *h* = 0.10 nm on the top electrode; Structure II, single protrusion with *r* = *h* = 0.50 nm; Structure III, dual protrusions with *r* = *h* = 0.25 nm on both electrodes (symmetric tip-to-tip). (**d**–**f**) QHT scattering spectra under a gap scan from 0.8 nm to 0.1 nm for Structures I–III, showing a red-to-blue crossover with turning points near *gap* = 0.6 nm (I), 0.3 nm (II), and 0.2 nm (III). The dashed arrows indicate the change trends of the peak positions, and different colors of lines indicate the gap sizes varied from 0.8 nm to 0.1 nm. (**g**–**i**) Representative QHT EF maps at *gap* = 0.1 and 0.6 nm, illustrating geometry-dependent persistence or suppression of gap localization at ultranarrow separations. For Structure I, the maximum EF decreases from 900 (*gap* = 0.6 nm) to 500 (*gap* = 0.1 nm). For Structure II, the maximum EF remains approximately constant at about 1200. For Structure III, the maximum EF increases from 900 to 2000, with strong localization maintained between the protrusion.

Having established the gap-driven crossover for a fixed protrusion, we next use QHT to determine how the turning-point gap depends on protrusion geometry. Figure 5 compares three representative junction configurations while scanning the gap. Structure I (Figure 5a) has a single small protrusion on the top electrode (radius and height both 0.10 nm); Structure II (Figure 5b) enlarges the single protrusion (radius and height both 0.50 nm); Structure III (Figure 5c) places protrusions on both electrodes (radius and height both 0.25 nm), forming a symmetric tip-to-tip junction. Figure 5d presents the QHT scattering spectra for Structure I under a gap scan from 0.8 nm to 0.1 nm. The dominant resonance again evolves non-monotonically, with a redshift upon initial compression followed by a blueshift at smaller gaps; the turning point appears near a gap of about 0.6 nm for this geometry. Figure 5e shows the corresponding spectra for Structure II. The same red-to-blue evolution is preserved, but the turning point shifts to smaller separations, around 0.3 nm, indicating that increasing the protrusion size delays the crossover to a more compressed junction. Figure 5f reports the spectra for Structure III (dual protrusions), where the turning point is pushed further still, to around 0.2 nm. Collectively, these results show that the turning-point gap is not universal; it is systematically tunable via protrusion geometry. The near-field distributions provide the real-space counterpart of this tuning. Figure 5g compares the enhancement maps for Structure I at *gap* = 0.6 nm (near the turning point) and *gap* = 0.1 nm. Near the turning point, the field is strongly confined within the junction; at 0.1 nm, the enhancement decreases significantly, the gap localization weakens, and the maximum EF drops from 900 to 500. Figure 5h shows the corresponding comparison for Structure II at *gap* = 0.6 nm and 0.1 nm. Here, the hotspot remains comparatively stable at 0.1 nm—consistent with the delayed crossover in Figure 5e—with a maximum EF of about 1200 in both cases. Figure 5i shows the corresponding maps for Structure III, where the enhancement at *gap* = 0.1 nm remains strongly concentrated within the ultrasmall region between the two protrusion apices, indicating that the symmetric tip-to-tip geometry sustains strong localization deeper into the sub-nanometer regime; correspondingly, the maximum EF increases from 900 to 2000.

Figure 4 and Figure 5 therefore identify the red-to-blue crossover and the concomitant non-monotonic near-field response as QHT-only signatures that are absent in the local classical description. At fixed geometry (Figure 4), compression initially strengthens confinement (redshift with increasing enhancement), but below a critical gap the response reverses (blueshift with suppressed enhancement). Extending to multiple geometries (Figure 5), the turning-point gap is strongly geometry dependent, shifting from about 0.6 nm (small single protrusion) to about 0.2 nm (symmetric dual protrusions), establishing protrusion design as a direct handle to tune the gap scale at which quantum-regulated, tunneling-relevant optical signatures emerge.

## 4. Conclusions

Atomic-scale protrusions and sub-nanometer separations are no longer rare imperfections in plasmonic junctions; they are increasingly the regime that sets device functionality. In this work, we use QHT to resolve how atomistic asperities reshape light–matter interaction in metallic nanogaps, and we benchmark the predictions against the LRA. First, we find that self-similar variations in an atomic protrusion leave the far-field scattering spectrum nearly unchanged, yet they strongly reconfigure near-field nanofocusing in both amplitude and spatial confinement. By separating aspect ratio from local curvature, we identify two competing controls of extreme nanofocusing: geometry concentrates charge toward the apex, whereas nonclassical electron response redistributes induced charge and current over a finite interfacial region, limiting hotspot collapse. This competition produces qualitative signatures that are absent in LRA. Second, upon gap compression, QHT predicts a robust red-to-blue crossover accompanied by a non-monotonic near-field evolution and a suppression of enhancement at ultranarrow separations, whereas LRA yields a purely monotonic redshift and unbounded field growth. Together, these results imply that deep in the sub-nanometer regime, LRA can be qualitatively misleading for both spectral trends and hotspot estimates. Finally, by scanning the gap across different protrusion geometries, we show that the tunneling signature is not universal but can be systematically tuned by morphology, with symmetric tip-to-tip junctions sustaining extreme localization deeper into the ultrasmall-gap regime. These findings convey a practical message for quantum plasmonics: far-field stability does not imply near-field stability once the junction enters the sub-nanometer regime. More broadly, they place atomic-scale morphology on the same footing as global antenna geometry for controlling optical energy concentration.

## Data Availability

The original contributions presented in this study are included in the article. Further inquiries can be directed to the corresponding author.

## Abbreviations

The following abbreviations are used in this manuscript:

QHT	Quantum Hydrodynamic Theory
LRA	Local-Response Approximation
TD-DFT	Time-Dependent Density-Functional Theory
PML	Perfectly Matched Layers
EF	Enhancement Factor

## Appendix A. Functional Form in QHT

Here we report the expression for G[*n*] obtained by taking the functional derivative with respect to *n* [49],

$$\frac{\delta T_{s}^{\mathrm{TF}}}{\delta n}=\frac{1}{2}\left(E_{h}a_{0}^{2}\right){\left(3\pi^{2}\right)}^{2/3}n^{2/3},$$

$$\frac{\delta T_{s}^{W}}{\delta n}=\left(E_{h}a_{0}^{2}\right)\frac{1}{8}\left(\frac{\nabla n\cdot \nabla n}{n^{2}}-2\frac{\nabla^{2}n}{n}\right),$$

$$\frac{\delta E_{\mathrm{XC}}^{\mathrm{LDA}}}{\delta n}=\left(E_{h}\right)\left(-a_{0}{\left(\frac{3}{\pi }\right)}^{1/3}n^{1/3}+\mu_{C}[n]\right)=v_{\mathrm{XC}}(r),$$

with

$$\mu_{C}[n]=\frac{-0.1423-0.1748\sqrt{r_{s}}-0.2381r_{s}}{{\left(1+1.0529\sqrt{r_{s}}+0.3334r_{s}\right)}^{2}}.$$

Here, *r_s_* = (3/4*πn*)^1/3^/*a*_0_. The explicit expressions of viscoelastic tensor are given below [46]:

$$\sigma =f_{\mathrm{CV}}\left(\frac{\partial v_{\alpha }}{\partial r_{\beta }}+\frac{\partial v_{\beta }}{\partial r_{\alpha }}-\frac{2}{3}\delta_{\alpha \beta }\nabla \cdot v\right),$$

where **v** = **P**/*n*_0_ is the velocity field of the conduction electrons, *f*_CV_ is the Conti-Vignale interpolation function [46], and *δ_αβ_* is the Kronecker delta function. The subscripts *α* and *β* denote Cartesian components [46].

$$f_{\mathrm{CV}}=\hbar n/\left(60r_{s}^{-2/3}+80r_{s}^{-1}-40r_{s}^{-2/3}+62r_{s}^{-1/3}\right).$$
